# On-axis structured beams generation via moiré and Mie resonant metallo-dielectric moiré gratings

**DOI:** 10.1038/s41598-025-01222-9

**Published:** 2025-05-13

**Authors:** Anil Ringne, Subrata Karmakar, Ananth Krishnan

**Affiliations:** Department of Electrical Engineering, Indian Institute of Technology Madras, Chennai, 600036 India

**Keywords:** Zeroth order, On-axis, Diffractive gratings, Logically flattened grating, Complex beams, Moiré pattern, Mie resonance, Plasmonic gratings, Nanophotonics and plasmonics, Sub-wavelength optics, Optical manipulation and tweezers

## Abstract

Structured beams carrying orbital angular momentum have been generated conventionally using spiral phase plates, fork gratings, and metasurfaces. Spiral phase plates are non-planar, fork gratings do not produce structured beams on the axis, and metasurfaces need subwavelength unit cell level design. In this work, we show a method to generate on-axis structured beams, at the zeroth order of a diffraction grating with experimentally relevant efficiency using moiré patterned binary gratings that are compatible with planar fabrication, do not need subwavelength unit cell level design, and can be used with a spatial light modulator. By logically superposing two binary forked gratings, we create a moiré pattern that enables on-axis structured beam generation at the zeroth order of the diffraction grating. We demonstrate, using experiments and simulations, the generation of on-axis zeroth order structured beams using spatial light modulator based reconfigurable moiré gratings and Mie resonant metallo-dielectric standalone moiré gratings, showcasing the versatility of this approach in different configurations. Simulations and experiments demonstrate that the on-axis structured beam is generated by the moiré pattern within the gratings, and its shape is determined by the topological charges of the overlapping binary forked gratings. Additionally, we demonstrate color-selective on-axis structured beam generation at the zeroth order of the grating, where the color-selectivity of the on-axis structured beam depends on the grating period and arises due to Mie resonance in standalone nanofabricated metallo-dielectric moiré gratings. The on-axis structured beam generation at the zeroth order of the grating using the proposed method may have several applications, including sensing and optical trapping.

## Introduction

Structured beams carrying orbital angular momentum (OAM) with multi-singularities^1^ have garnered significant interest for their structured phase profile with the simultaneous existence of numerous OAM modes, which enable progress in sensing^2,3^, particle trapping^4–10^, information coding^11^, and communication^12–16^.

To realize these structured beams, researchers have explored techniques such as spiral phase plates^17–20^, fork gratings^2,21,22^, and metasurfaces^12,23,24^. Spiral phase plates introduce an azimuthally varying optical path difference created using azimuthal thickness variation of material, imparting the desired structured phase profile to an incident beam. Fabricating such a non-planar structure needs quantization of the material thickness variation, requiring variable dose electron beam lithography or precise etching techniques, making the process complicated and challenging to scale for different wavelengths^18,20,25^.

Alternatively, fork gratings can generate structured beams by employing the principle of diffraction, achieved through the combination of a tilted plane and the helical phase fronts^21,22,26^. Their fabrication is advantageous because they can be implemented as binary planar structures using standard lithography techniques with less demanding fabrication tolerances, ensuring scalability across different wavelengths and sizes^2^. However, the structured beams generated from fork gratings appear in the first diffraction orders rather than the zeroth order of the grating. As a result, their use in in-plane applications such as microscopy and imaging is limited, and the experimental setup becomes more intricate and bulky^22,26^. Metasurfaces, on the other hand, as planar optical devices, leverage subwavelength structures to precisely tailor phase and amplitude at the nanoscale, enabling the generation of structured beams^12,23,24^. Despite their capabilities, metasurfaces face critical challenges in unit cell parameter optimization, stringent fabrication tolerances, and a lack of reconfigurability, at least in the visible wavelengths^24^. Q-plates and spatial light modulators are well-established tools for generating structured beams with vortices in collinear configurations. In fact, part of the present work employs SLMs to experimentally validate the beam shaping concept. However, the generation of structured beams carrying vortices at the zeroth order of a grating is of significant interest in fields involving collinear microscopy-based optical investigations and plasmonic microscopy. In such microscopy, the device sizes will be around a few hundred micrometers. In such a confined geometry, conventional devices like q-plates and SLMs, which typically operate over millimeter-scale apertures and require free-space beam propagation or large optical setups, are not practical for generating structured beams at the zeroth order. Thus, the use of nanostructured gratings provides a unique and compact alternative, enabling direct integration with high-resolution microscopy systems while maintaining collinearity and minimizing alignment complexities.

To address these challenges, the present manuscript introduces a novel approach that incorporates moiré patterns into binary gratings, enabling the generation of an on-axis structured beam at the zeroth order of a grating. Various types of gratings can be used to form moiré pattern^27,28^ which usually employed to analyze small-scale structural misalignments^29–31^, study periodic or quasi-periodic structures^32–34^, and enhance imaging resolution^35–37^. We logically overlap two binary forked gratings to form a moiré pattern yielding at a larger period due to the interaction of their spatial frequencies, which eventually produces the on-axis structured beam at the zeroth order of a grating. The on-axis structured beam generated at the zeroth order of the grating, with the grooves providing the ‘$\pi$’ phase, is estimated to have an efficiency of 12%, making it experimentally relevant. The proposed method is first validated using simulation, and then spatial light modulator-based experiments are used to demonstrate the on-axis zeroth order structured beam generation. We establish that the shape of the on-axis zeroth order structured beam is governed by the topological charges of the overlapped binary forked gratings.

However, the on-axis zeroth order structured beam has a direct light beam component (DC component). A crossed polarization microscopy technique is used to mitigate this DC component by incorporating a resonance into the grating. A crossed polarization microscopy technique can mitigate the DC components from the on-axis zeroth order structured beam with a wide working wavelength range. However, the grating should be resonant. To address this, the Mie resonance is incorporated in logically flattened gratings using fabricated standalone metallo-dielectric moiré gratings. In reflection, we generated the color-selective on-axis structured beams at the zeroth order of a grating using fabricated standalone metallo-dielectric moiré gratings. The color-selective characteristics of zeroth-order structured beams imply that the gratings are resonant and could be used to mitigate the DC component in a crossed polarization microscopy arrangement. We demonstrate that the on-axis structured beam’s color selectivity is primarily controlled by the grating period through Mie resonance effects. Overall, we show a DC component free on-axis structured beam generation at the zeroth order of a grating via standalone and reconfigurable gratings, which may have several applications, including sensing and optical trapping.

## Main

**Fig. 1:**
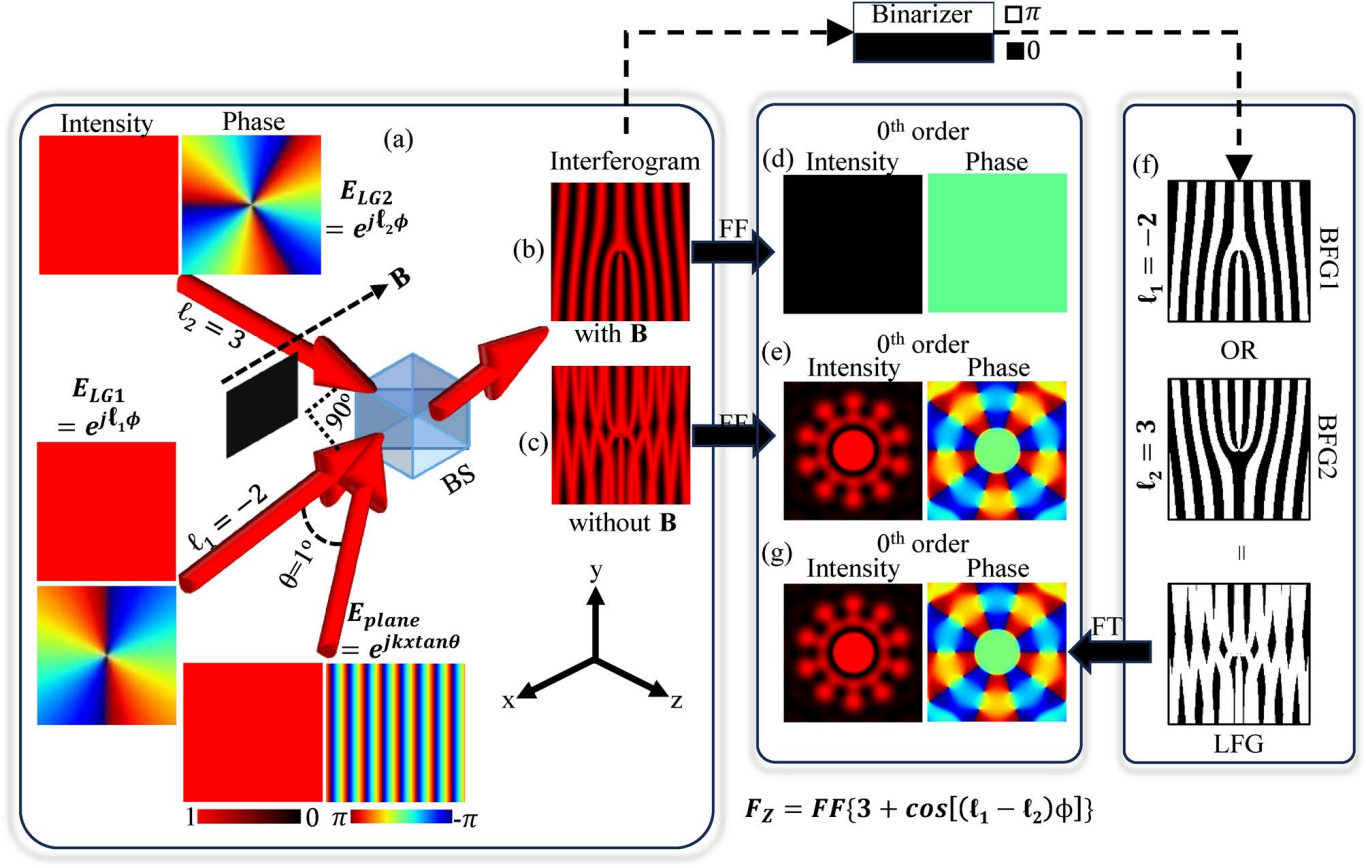
(**a**) Interferometry setup to generate the forked-shape and planar interferograms, referred to as the forked grating and flattened grating, respectively. (**b**) designed forked grating of $\ell =-2$ when Blocker B blocked the Laguerre–Gaussian LG2 beam, (**c**) designed flattened grating of $\ell =-2$ and 3, (**d**) zeroth order of 2D Fourier transform of forked grating of $\ell =-2$, (**e**) zeroth order of 2D Fourier transform of flattened grating of $\ell =-2$ and 3, (**f**) designing of a Logically Flattened Grating LFG using Binary Forked Gratings BFG of charges $\ell _1=-2$ and $\ell _2=3$, and (**g**) zeroth order of 2D Fourier transform of designed LFG. The phase of the incident plane wave is expressed as $e^{j k x \tan \theta }$, corresponding to a beam propagating at an angle $(\theta )$ relative to the optical axis of the LG1 beam. This formulation captures the transverse phase gradient introduced by the angular tilt and accurately represents the incident field in the interferometric setup. A tilted plane wave with a tilt angle of $1^o$ is considered to design the gratings.

A unity amplitude Laguerre Gaussian (LG) beam LG1 interferes with a unity amplitude tilted plane wave, forming a forked grating. The interference between these beams can be achieved using the interferometric setup shown in Fig. 1a, while a Blocker (B) is enabled to block the path of the second unity amplitude LG (LG2) beam. The sketch of the forked-shape interferogram is shown in Fig.  1b, hereafter referred to as the forked grating. This forked grating has a central discontinuity equal to the topological charge of the LG1 beam. Similarly, the combination of LG1, LG2, and tilted plane waves (upon disabling the LG2 blocker, B) leads to the formation of a planar interference pattern, hereafter referred to as the flattened grating, as depicted in Fig. 1c. When two or more gratings with a variation in amplitude from 0 to 1 are overlapped, and the resultant grating formed using maximum or OR logical operation on gratings will also have an amplitude variation from 0 to 1, then this process is referred to as flattening of the grating. The flattened grating can be expressed mathematically in terms of the electric fields of LG1, LG2, and tilted plane waves. The electric field ($E_{plane}$) of unity-amplitude tilted plane wave with tilt angle ($\theta$) with respect to the *x*-axis is given by Eq. 1 in phasor form^38,39^:

1 $$\begin{aligned} E_{plane} = e^{j k x \tan \theta }, \end{aligned}$$

In Eq. 1, wave number (*k*) is given by $k=2 \pi / \lambda$, and $\lambda$ is the wavelength of the beams. Similarly, the electric fields ($E_{LG1}$ and $E_{LG2}$ ) of unity amplitude LG beams with topological charges $\ell _1$ and $\ell _2$ respectively, are given by Eqs. 2 and 3 with angular coordinates ($\phi$):

2 $$\begin{aligned} & E_{LG1} = e^{j \ell _1 \phi }, \end{aligned}$$

3 $$E_{{LG2}} = e^{{j\ell _{2} \phi }} ,$$

The Intensity (I) of the flattened grating (interferogram obtained by interfering the two unity amplitude LG beams, LG1 and LG2, with unity amplitude tilted plane wave) is written according to the interference formulation^40^, shown by Eq. 4:

4 $$\begin{aligned} I= (E_{plane} + E_{LG1} + E_{LG2}) \times (E_{plane} + E_{LG1} + E_{LG2})^*. \end{aligned}$$

Equation 5 is the equation for the flattened grating which can be obtained by simplifying Eq. 4 using Eqs. 1 to 3:

5 $$\begin{aligned} I= \textbf{3} \pmb {+} \textbf{2} \pmb {\cos [(\ell _1-\ell _2) \phi ]}+2 \cos [\ell _1 \phi - k x \tan (\theta )]+2 \cos [\ell _2 \phi - k x \tan (\theta )]. \end{aligned}$$

In Eq. 5, $(2 \cos [\ell _1 \phi - k x \tan (\theta )]+2 \cos [\ell _2 \phi - k x \tan (\theta )])$ terms contain wave number ‘k’ and ‘tan($\theta$)’, which represent the higher order structured beam generation using a grating^22^. Whereas, the $(\textbf{3} \pmb {+} \textbf{2} \pmb {\cos [(\ell _1-\ell _2) \phi ]})$ term consists of a pure DC term of ‘3’ and an $\ell _1$ and $\ell _2$ dependent cosine term that is free of wave number ‘k’. Hence, the structured beam can be expected in the zeroth order of the grating. A 2D matrix representing the grating was symmetrically padded to increase the aperture, and the Fourier transform was computed using numpy.fft.fft2 in Python. To center the zero-frequency component, we used numpy.fft.fftshift, following standard practice for spatial frequency analysis. Visual representations of the Fourier transform of the forked grating and the $(\textbf{3} \pmb {+} \textbf{2} \pmb {\cos [(\ell _1-\ell _2) \phi ]})$ term at zeroth order are shown in Fig. 1d and e, respectively. A bright spot, along with the flower-like pattern composed of several nulls is observed in the zeroth order diffraction pattern of the flattened grating. As this flower-like diffraction pattern is also in line with the zeroth order bright spot, it is referred to as an on-axis structured beam. In the case of the forked grating, no diffraction pattern is observed due to no modulation at the zeroth order.

Similar to the flattened grating, a Logically Flattened Grating (LFG) can be used to generate an on-axis structured beam, which is a binary version of the flattened grating. For simplicity, two Binary Forked Gratings (BFG), BFG1 and BFG2, can be logically overlapped to form an LFG. Individual BFGs (BFG1 and BFG2) can be created by binarizing the interferogram of individual unity amplitude LG beams and tilted plane wave, as an example shown by the transformation from Fig. 1b to f. The created LFG can be used as an amplitude grating as well as a phase grating. Hence, it can be utilized as a single standalone component to generate on-axis structured beams. Any logical operation can be used to generate structured beams^22^. However, OR logical operation is used here for simplicity.

All the BFGs and LFGs are shown with white and black color regions. The white and black regions of the gratings provide ‘$\pi$’ and ‘0’ phase modulation, respectively, to the input beam. The width of sum of a white region and a black region is called the period ($\Lambda$) of the grating. Thus, the Duty cycle (D) is given by the ratio of the width of a white region to the $\Lambda$ of the grating. For the simple case, a duty cycle of 50% is considered to create BFGs. A visual representation of the LFG and its Fourier transform are shown in Fig. 1f and g, respectively. An on-axis structured beam generated by LFG is similar to the on-axis structured beam generated by a flattened grating, as can be observed.

**Fig. 2:**
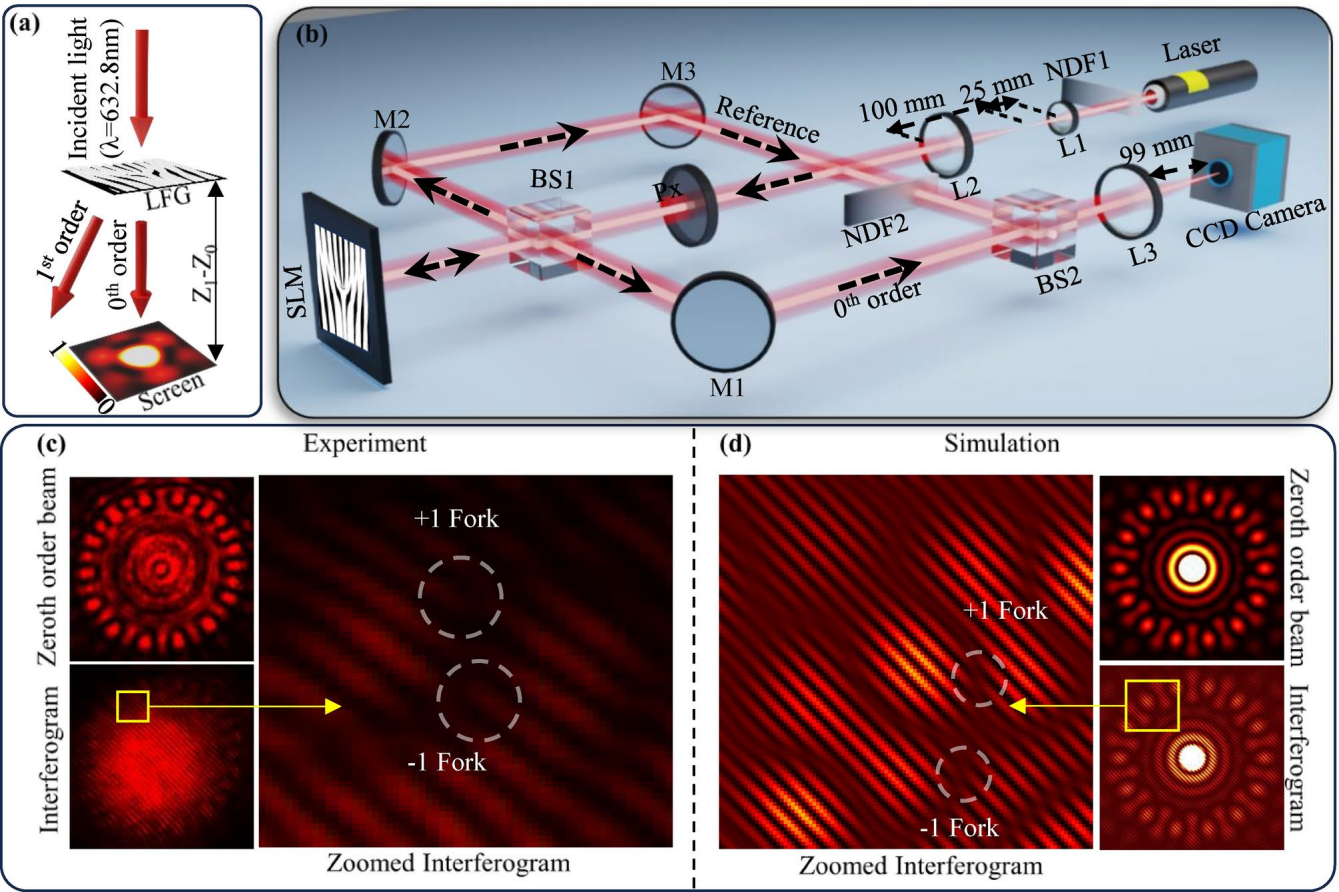
(**a**) Schematic of Huygens’ principle based simulation setup, (**b**) spatial light modulator based experimental setup for generation of on-axis zeroth order beams, (**c**) experimental and (**d**) simulated interferogram and zeroth order beam profiles of LFG of $\ell =7$ and $-7$.

In order to validate the concept of on-axis structured beam generation, initially, a Huygens’ principle based simulation setup and a Spatial Light Modulator (SLM) based experimental setup are used. In the Huygens principle based simulation setup, each point of the grating acts as a spherical source, and the interference of these sources is captured on the screen, as shown in Fig. 2a. Each white and black point of the grating of size ${\text{P}}_x \times$ ${\text{P}}_y$ spatially modulates the phase of the incident electric field $E(x_0, y_0, z_0)$ by ‘$\pi$’ and ‘0’, respectively. The sum of electric fields from these sources on the screen can be expressed as $E(x_1, y_1, z_1)$, which is given by Eq. 6:

6 $$\begin{aligned} E(x_1,y_1,z_1) = -\frac{j(z_1-z_0)}{\lambda } \sum _0^{P_x} \sum _0^{P_y} \frac{E_0(x_0, y_0, z_0) \exp \left( -j \frac{2\pi }{\lambda }r\right) }{r^2} \Delta x \Delta y. \end{aligned}$$

The $(x_0, y_0, z_0)$ and $(x_1, y_1, z_1)$ are the coordinates of phase modulated spherical sources and the summation point on the screen, respectively. The r is the distance between each spherical source and each point of the screen, calculated by $r=\sqrt{(x_1-x_0)^2+(y_1-y_0)^2+(z_1-z_0)^2}$. $\Delta x$ and $\Delta y$ are the distance between two adjacent sources on the grating in the x and y directions, respectively. For simulations, a plane wave of 632.8 nm wavelength, a grating of size 82 $\upmu$m $\times$ 82 $\upmu$m with 1024 $\times$ 1024 grating points, a period of 960 nm, a $z_0$ of 0, and a $z_1$ of 1.5 mm are used.

The SLM based experiments are conducted using the setup shown in Fig. 2b. In the shown experimental setup, a He-Ne laser with a wavelength of 632.8 nm is used to generate structured beams. The laser intensity is adjusted as per the SLM requirements using a neutral density filter, NDF1. The adjusted beam is expanded using two convex lenses, L1 of 50 mm and L2 of 100 mm focal lengths. A horizontal polarizer Px is used to polarize the incident expanded beam. The polarized beam was incident on 8-bit PLUTO-2-VIS-016 phase-only SLM via beam splitter, BS1. The binary grating images were loaded onto the SLM, with the white and black points of the grating mapped to $\pi$ and 0 phase modulation, respectively, as per the SLM’s phase-pixel calibration. BS1 is used to generate a reference beam for interference, and it is also used to reflect the diffracted beams from SLM toward the Charge Coupled Device (CCD) camera. A mirror M1 and convex lens L3 of 100 mm focal length were used for proper alignment and focussing of diffracted beams on the CCD camera. A Mach-Zehnder interferometric setup was constructed using two mirrors, M2 and M3, and two beam splitters, BS1 and BS2. The intensity of the reference beam is adjusted using a second neutral density filter, NDF2. The intensity matched reference beam interfered with the zeroth order diffracted beam and generated the interferograms. The same CCD camera also captured the generated interferograms. The 8.2 mm $\times 8.2$ mm gratings having a 96 $\upmu$m period and $1024 \times 1024$ points are considered for the experiment.

Figure 2c shows the experimentally captured on-axis zeroth order beam pattern and interferogram for LFG of $\ell _1=7$ and $\ell _2=-7$. A central bright spot surrounded by twenty eight petals or vortices can be observed experimentally in the captured on-axis intensity pattern. When a tilted plane wave interferes with a vortex, a forked shaped interferogram is formed. Hence, the formation of $+$ 1 and − 1 topologically charged forked patterns observed in the interferogram, suggesting the formation of $\ell =1$ and $\ell =-1$ vortices simultaneously between each petal of the on-axis structured beam. These vortices are closely spaced, hence the intensity between the vortices is lowered. However, the phase singularities (forked patterns in the interferogram) are still intact. This suggests the formation of structured beams in the zeroth order of the grating.

For a similar LFG of $\ell _1=7$ and $\ell _2=-7$, the simulated on-axis zeroth order beam pattern and interferogram are obtained, shown in Fig. 2d. The simulated on-axis structured beam with a central bright spot surrounded by twenty-eight petals or vortices is observed to be matched with the experimental on-axis structured beam. However, a slight discrepancy is observed mainly due to the pixelation of the SLM and the aperture effect of the gratings^22^. The pixelation of SLM affects the overall duty cycle of the grating, causing the lower intensity in the zeroth order^22^. Similar to the experiment, in simulations the forked shape patterns of topological charges $\ell =1$ and $\ell =-1$ can be observed in the interferogram which suggest the formation of $\ell =1$ and $\ell =-1$ vortices simultaneously but closely spaced, which also concludes the generation of an on-axis structured beam at the zeroth order of the LFG.

**Fig. 3:**
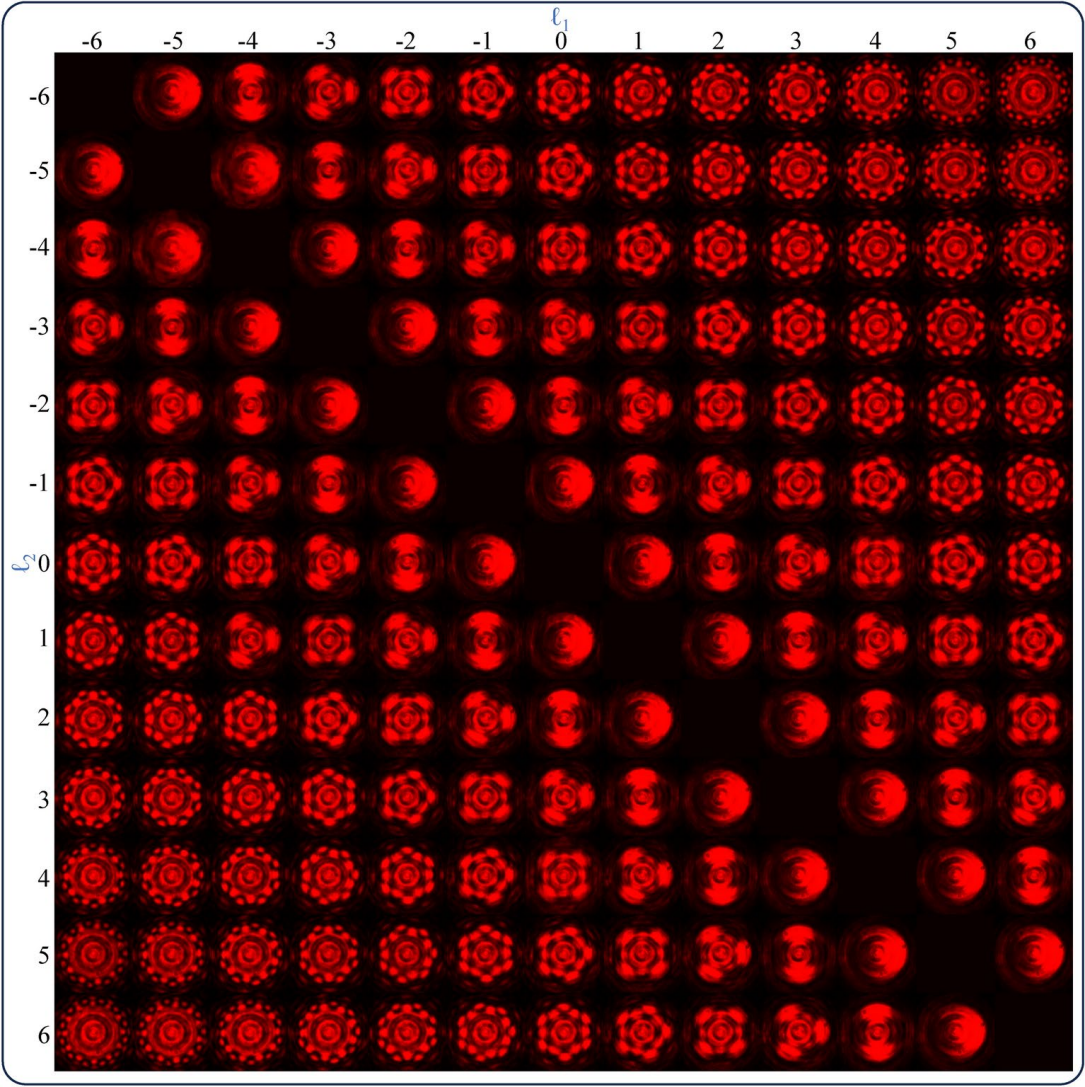
Experimentally obtained on-axis structured beams for LFGs of different azimuthal charge combinations.

Figure 3 shows the experimentally obtained on-axis intensity patterns of LFGs created by OR logical flattening of two BFGs, BFG1 of charge $\ell _1$ and BFG2 of $\ell _2$. Both $\ell _1$ and $\ell _2$ are varied from − 6 to + 6, and the on-axis intensity patterns are captured with the same exposure settings. For $\ell _1 \ne \ell _2$, the generation of an on-axis structured beam can be observed, which has a central spot surrounded by $2|\ell _1-\ell _2|$ petals/vortices. Hence, the lowest possible number of petals/vortices is 2 for the integer values of charges. The size of the on-axis intensity pattern is observed to be increasing with $|\ell _1-\ell _2|$, as expected^22^. As the LFGs are created by OR logical flattening of two BFGs, the $\ell _1$ and $\ell _2$ are interchangeable. Hence, a symmetry across the diagonal of the figure is observed. For $\ell _1=\ell _2$, there is no zeroth order intensity profile because, for $\ell _1=\ell _2$, the created LFG acts as a regular fork grating.

**Fig. 4:**
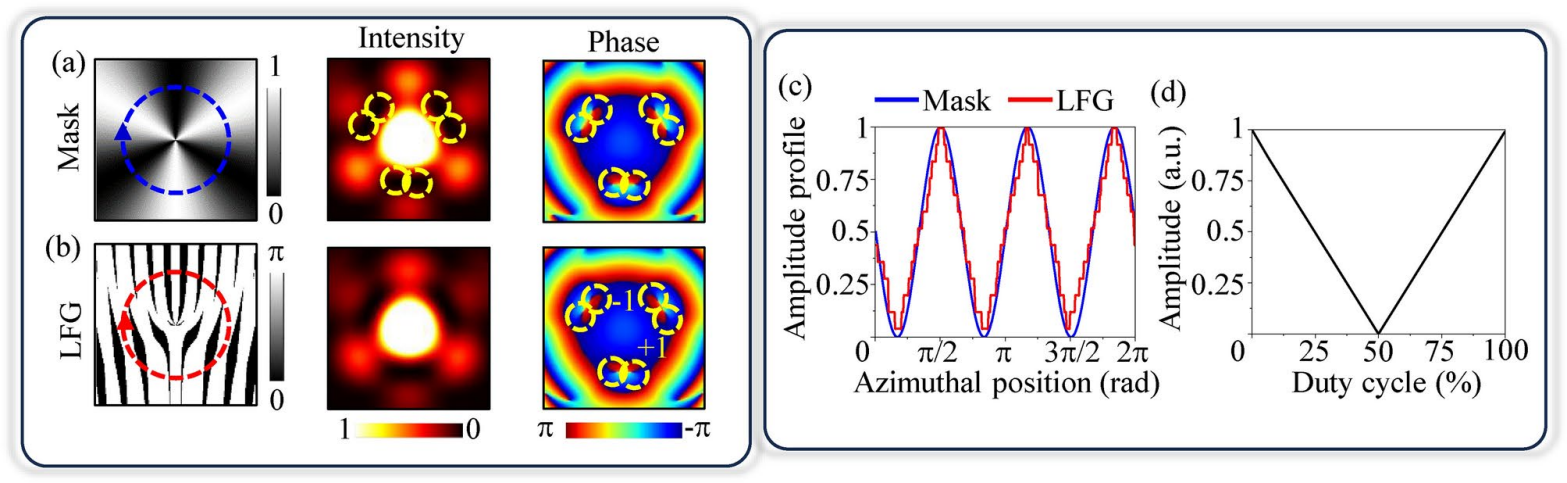
Comparison of simulated intensity and phase pattern of on-axis structured beam obtained using (**a**) amplitude mask, and (**b**) LFG, (**c**) azimuthal amplitude profile of the mask and LFG, (**d**) graph of zeroth order amplitude profile with the variation in the duty cycle of the grating.

Figure 4a and b show the amplitude mask and the LFG of charges 2 and 5, respectively, along with their simulated far field on-axis intensity, and phase pattern. By comparing far field intensity and phase patterns of amplitude mask and LFG, it can be observed that the on-axis structured beam is generated due to azimuthal amplitude variation present in the grating, as explained in Eq. 5, and using simulations and experiments in Fig. 2. The yellow dotted circles in intensity and phase patterns show the closely spaced + 1 and − 1 charge vortices, simultaneously present in the on-axis structured beam, as expected. When two BFGs are logically flattened, a moiré pattern is formed due to the local variation of the duty cycle in the azimuthal direction. The variation in the duty cycle causes the local change in amplitude profile of the grating^22^. The amplitude profiles of the mask and LFG in the azimuthal direction are shown in Fig. 4c. The variation in the duty cycle of LFG is mapped with the simulated zeroth order amplitudes, as shown in Fig. 4d. From Fig. 4c, it can be observed that the amplitude profiles of the mask and LFGs in azimuthal direction are nearly matched. This clearly suggests the zeroth order structured beam generation is indeed from the moiré pattern formed by the LFGs. In addition, the structured beams such as radial carpet beams^41^, Airy beams^41–43^, and HG beams are different than the generated zeroth order structured beam by phase patterns and/or by intensity patterns. The generated structured beams have phase singularities in the periphery of the beam. Whereas, the radial carpet beams generated via radial phase gratings do not show any singularities in the phase pattern. Airy beams and HG beams usually do not carry any phase singularities either. To have the vortex in these beams, specially created gratings can be used^22^.

**Fig. 5:**
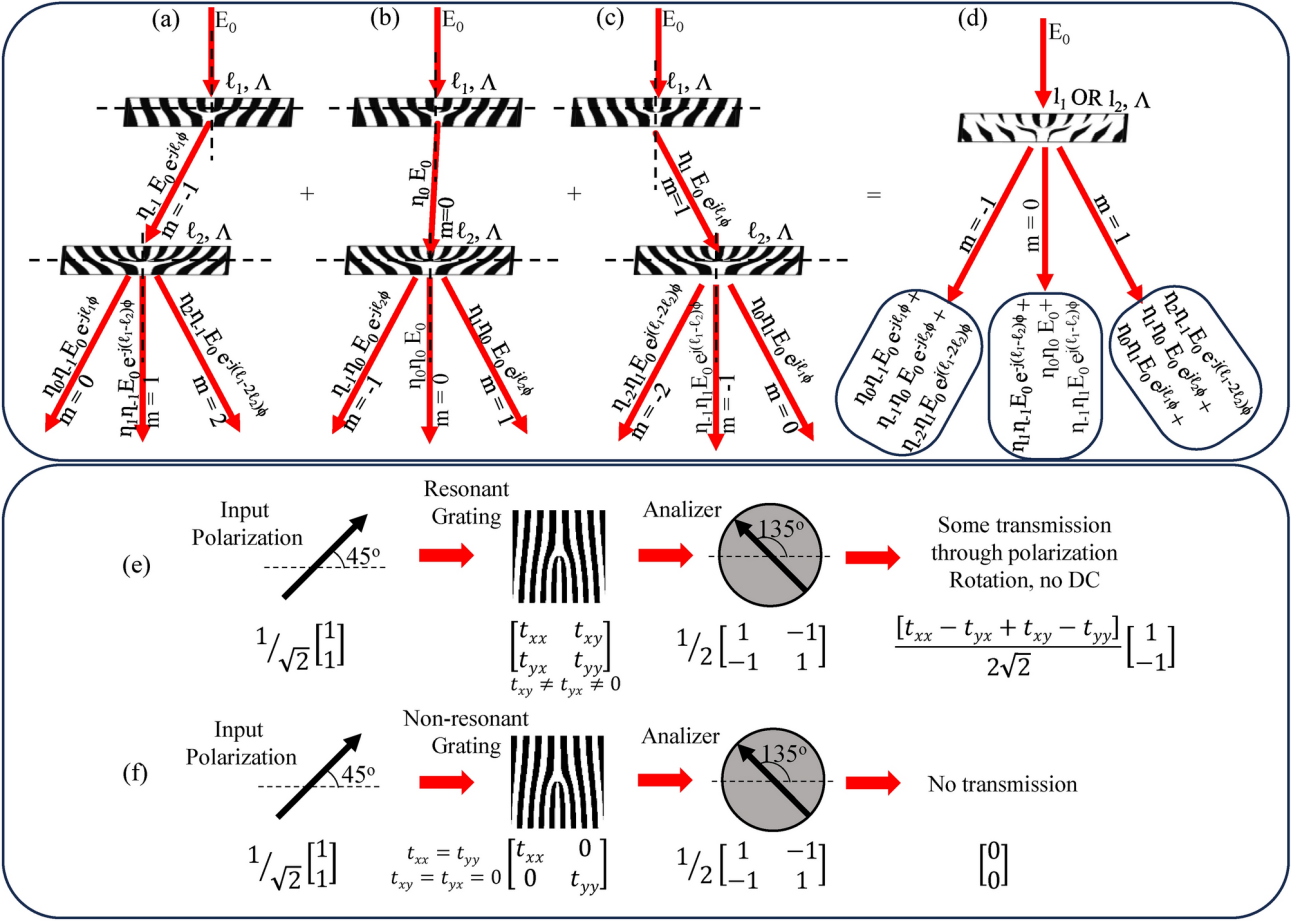
(**a**)–(**d**) Calculation of superposition of Laguerre–Gaussian (LG) modes. A tilted view of two overlapping gratings considering one forked grating BFG1 on top of another, whose (**a**) m = − 1, (**b**) m = 0, and (**c**) m = 1 order beams interact with another forked grating, BFG2. (**d**) A tilted view of resultant LFG and its resultant superposition of LG beams in − 1st, 0th, and 1st order. Jones vector calculation in the crossed polarization microscopy technique in case of (**e**) resonant and (**f**) non-resonant gratings.

In addition, the LFGs can be treated as two binary forked gratings, BFG1 and BFG2, one over the other, as shown in Fig. 5. When a light beam is incident on a BFG with topological charge $\ell$, the generated LG beams in the *m*-th diffraction order can be simply represented as having the phase term $e^{jm\ell \phi }$ and efficiency factor $\eta _m$ multiplied, though the actual beam structure can be more complex. Figure 5a shows the light of $E_0$ incident on the BFG1 generating $\eta _{-1} E_0 e^{-j\ell _1\phi }$ beam in the − 1st order of the grating. This $\eta _{-1} E_0 e^{-j\ell _1\phi }$ beam interacts with the BFG2 and generates $\eta _{0}\eta _{-1} E_0 e^{-j\ell _1\phi }$, $\eta _{1}\eta _{-1} E_0 e^{j(-\ell _1+\ell _2)\phi }$ and $\eta _{2}\eta _{-1} E_0 e^{j(-\ell _1+2\ell _{2})\phi }$ in 0th, 1st and 2nd diffraction order of the grating. Similarly, Fig. 5b shows the light of $E_0$ incident on the BFG1 generating $\eta _{0} E_0$ beam in the 0th order of the grating. This $\eta _{0} E_0$ beam interacts with the BFG2 and generates $\eta _{-1}\eta _{0} E_0 e^{-j\ell _2\phi }$, $\eta _{0}\eta _{0} E_0$ and $\eta _{1}\eta _{0} E_0 e^{j\ell _{2}\phi }$ in − 1st, 0th and 1st diffraction order of the grating. Likewise, Fig. 5c shows the light of $E_0$ incident on the BFG1 generating $\eta _{1} E_0 e^{j\ell _1\phi }$ beam in the 1st order of the grating. This $\eta _{1} E_0 e^{j\ell _1\phi }$ beam interacts with the BFG2 and generates $\eta _{0}\eta _{1} E_0 e^{j\ell _1\phi }$, $\eta _{-1}\eta _{1} E_0 e^{j(\ell _1-\ell _2)\phi }$ and $\eta _{-2}\eta _{1} E_0 e^{j(\ell _1-2\ell _{2})\phi }$ in 0th, − 1st and − 2nd diffraction order of the grating. The combination of these beams is shown as the output of LFG in Fig. 5d. In − 1st order of the LFG, the superposition of $\eta _{0}\eta _{-1} E_0 e^{-j\ell _1\phi }$, $\eta _{-1}\eta _{0} E_0 e^{-j\ell _2\phi }$ and $\eta _{-2}\eta _{1} E_0 e^{j(\ell _1-2\ell _{2})\phi }$ can be observed. As $\eta _{\pm 2}< \eta _{\pm 1}< \eta _{0}$, the term $\eta _{-2}\eta _{1} E_0 e^{j(\ell _1-2\ell _{2})\phi }$ can be neglected, and hence in the − 1st order of the LFG, the superposition of $\eta _{0}\eta _{-1} E_0 e^{-j\ell _1\phi }$ and $\eta _{-1}\eta _{0} E_0 e^{-j\ell _2\phi }$ can be observed, as expected^22^. Similary, in 1st order of the LFG, the superposition of $\eta _{0}\eta _{1} E_0 e^{j\ell _1\phi }$ and $\eta _{1}\eta _{0} E_0 e^{j\ell _2\phi }$ can be observed, as expected^22^. In 0th order, a complex pattern of superposition of $\eta _{1}\eta _{-1} E_0 e^{j(-\ell _1+\ell _2)\phi }$, $\eta _{0}\eta _{0} E_0$ and $\eta _{-1}\eta _{1} E_0 e^{j(\ell _1-\ell _2)\phi }$, can be observed.

As $\eta _{\pm 2}< \eta _{\pm 1}< \eta _{0}$, the term $\eta _{0}\eta _{0} E_0$ has maximum efficiency, and it is a DC term carrying no spatial information of gratings. To filter out this term, a resonant-based grating can be utilized along with a cross-polarizer-analyzer setup, as shown in Fig. 5e and f. As shown in Fig. 5e, a 45° polarized light, when interacting with the resonant grating, through scattering, the spatially affected beams undergo polarization rotation. However, DC terms are unaffected by grating and preserve the input 45° degree polarization. When DC terms and polarization-rotated beams interact with the 135° analyzer, DC terms are mitigated, due to the complementary analyzer oriented along 135° as per Malus’s law. Only polarization-rotated beams (zeroth order structured beams + ± first order structured beams) survive, as desired. Of course, the maximum intensity of output light will be by keeping the grating at 90°, as per Malus’s law. In the case of non-resonant grating, all the spatially affected beams and DC terms preserve the input 45° degree polarization and are mitigated by the 135° analyzer, as shown by Fig. 5f.

**Fig. 6:**
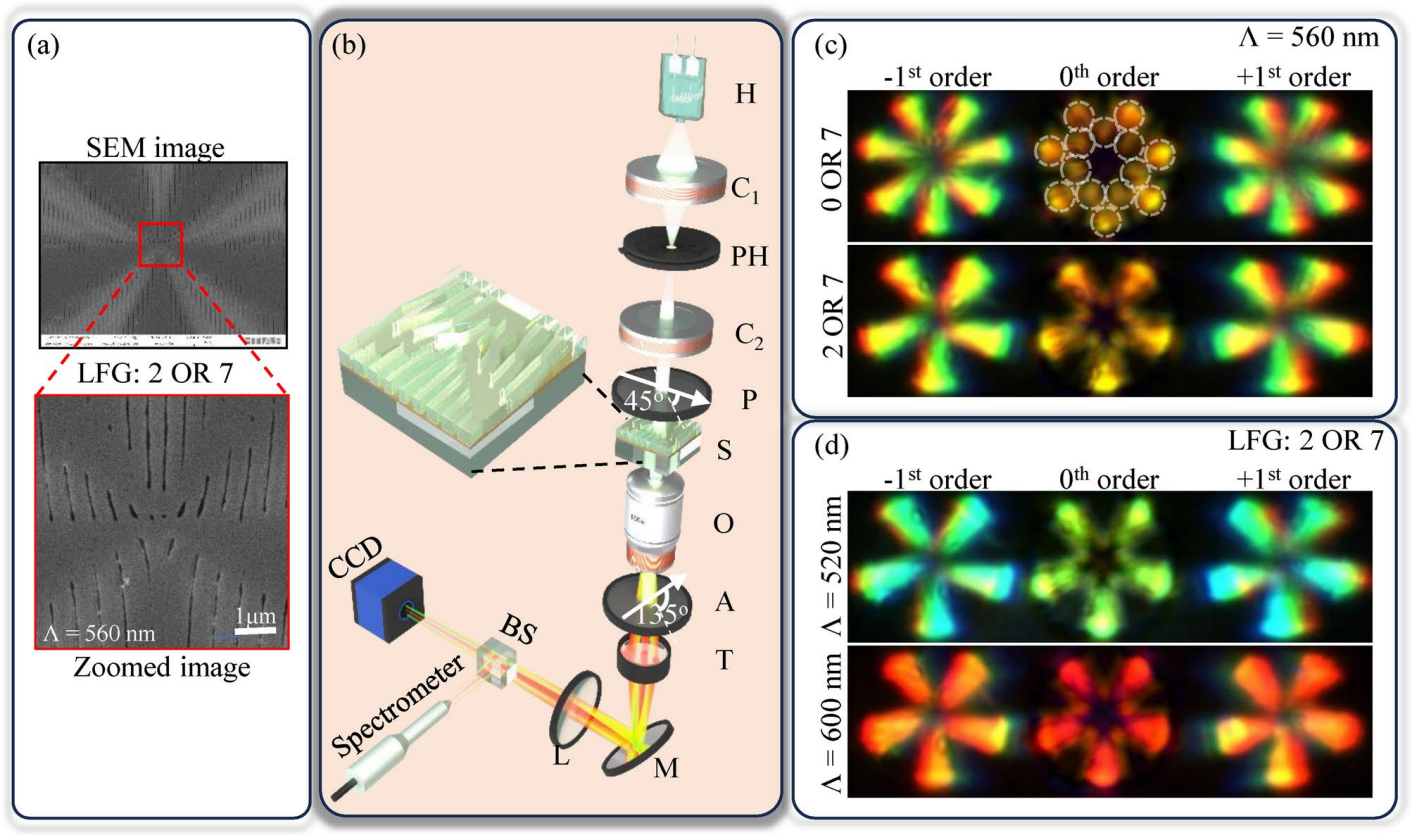
(**a**) Fabricated and zoomed image of metallo-dielectric LFG of charge 2 and 7 of period 560 nm, (**b**) experimental setup to obtain on-axis structured beam using fabricated structure under broadband illumination, (**c**) structured beams generated using different charge combinations of LFGs having a period of 560 nm. (**d**) structured beams generated using LFGs of charge 2 and 7 of different periods.

Polymethyl methacrylate (PMMA) LFGs over thin gold (Au) metal film are fabricated using the Electron beam (E-beam) lithography method. The fabricated grating is circular with a radius of $50\,\upmu \,\text {m}$, resulting in an effective area of approximately $7850\,\upmu \,\text {m}^2$ ($\approx \pi \times (50\,\upmu \,\text {m})^2$), which is fully utilized in the experiment. The fabricated metallo-dielectric LFG with topological charges (2 OR 7) is shown in Fig. 6a. LFG’s thickness was nearly 200 nm for near ‘$\pi$’ modulation to the input beam, similar to the initial experiments. However, a minimum experimentally possible Au film of 15 nm is deposited to ease fabrication. The fabricated LFG is characterized by the Nikon Ti E inverted microscope, whose schematic setup is shown in Fig. 6b. The microscope consists of a Broadband (B) source, Collector (${\text{C}}_1$) lens, Pin Hole (PH), Collimating (${\text{C}}_2$) lens, 100× Objective (O) lens, Tube (T) lens, and flip Mirror (M). The pure dc term $(\textbf{3})$ or ($\eta _{0}\eta _{0} E_0$) in the zeroth order of a grating shown in Eq. 5 is an unmodulated beam affecting the on-axis structured beams. In order to filter out this unmodulated beam, the crossed polarization microscopy technique^44^ is used. Hence, a pair of Polarizers (P) at 45° considering (TE+TM)/2 polarization configuration and Analyzer (A) at 135° are used, which also block the direct transmission from the source. The images are captured using a 100 mm focal length convex Lens (L) and a CCD camera. The polarized light is incident on the Sample (S) consisting of LFGs that generate the structured beams in + 1, 0, and − 1 order. The spectral response from S in various orders are recorded using a visible range spectrometer.

The generated structured beams for metallo-dielectric LFGs of two charge combinations (0 OR 7) and (2 OR 7) are shown in Fig.  6c. In + 1 and − 1 orders, as expected, structured beams with 7 and 5 petals for LFGs of charges (0 OR 7) and (2 OR 7) can be observed, respectively^22^. In addition, color selectivity in these structured beams at + 1 and − 1 orders can be observed. In zeroth order, a structured beam with 14 and 10 petals for LFGs of charges (0 OR 7) and (2 OR 7) can be observed, respectively. The number of petals in the zeroth order perfectly aligns with the expected outcomes. However, the central bright spot is diminished as desired due to polarization filtering by a pair of polarizers P and A (crossed polarization microscopy technique). In addition, the color selectivity is also observed in zeroth order structured beams. These color selectivities in the different orders of LFGs are shown in Fig. 6c and d by considering LFGs of charge (2 OR 7) with periods 520 nm, 560 nm and 600 nm. A red shift in both the orders is observed with an increase in the period of LFGs.

**Fig. 7:**
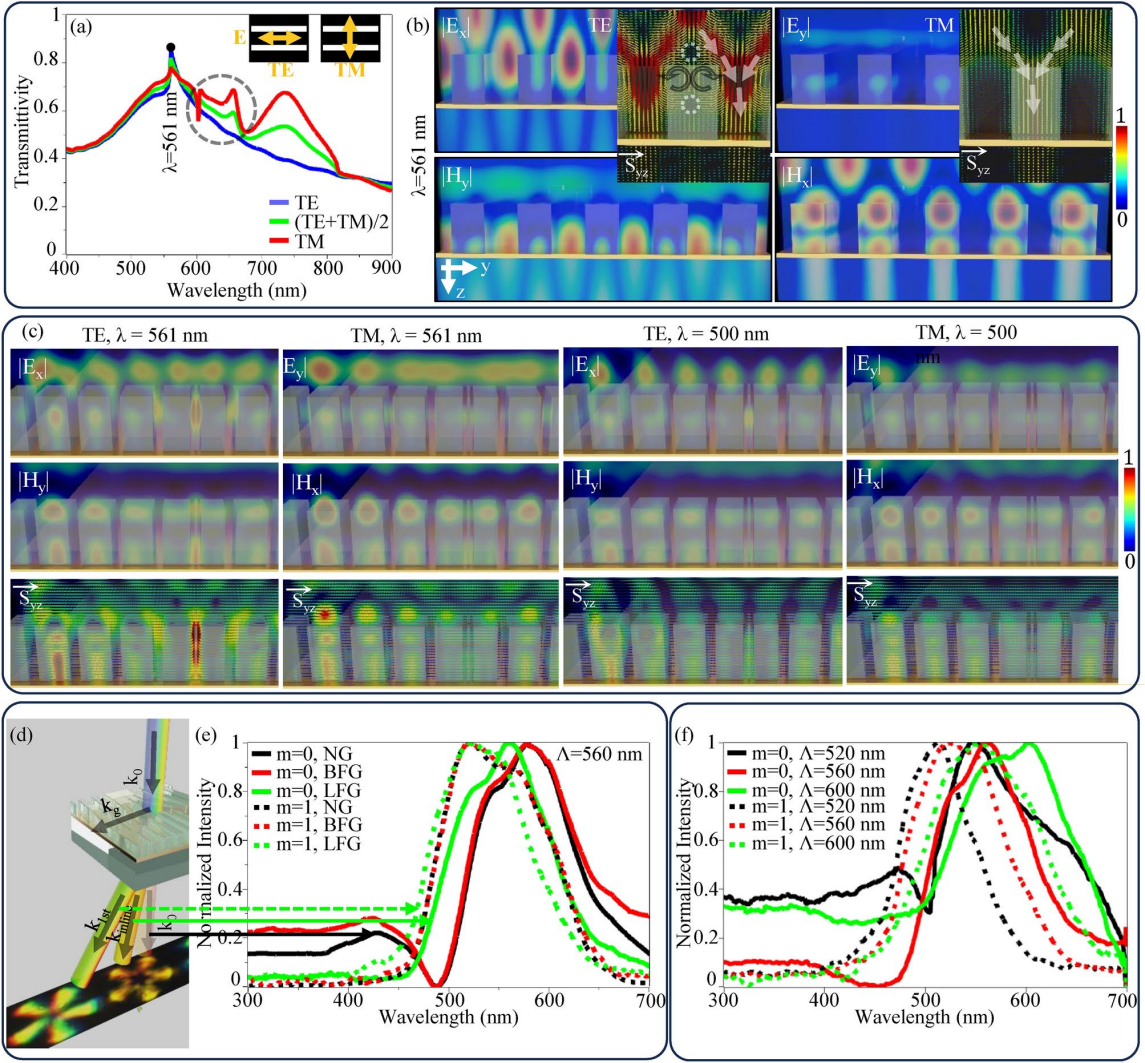
(**a**) Simulated transmittivity of Normal Grating NG under various polarizations, inset of the figure show the polarization definitions, (**b**) electric and magnetic field profiles at a resonant wavelength for Transverse Electric TE and Transverse Magnetic TM polarizations, inset images show the transverse Poynting vector diagram in yz plane where black and white arrows show the circulation and overall direction of transverse Poynting vectors, respectively, and white dotted circles show the saddle type singularity points (**c**) electric, magnetic and transverse Poyting vector diagrams for metallo-dielectric LFGs of charge 2 and 7 at wavelength 561 nm and 500 nm, (**d**) wave vector mapping and visual representation of spectral shifts in various orders, (**e**) experimentally obtained self normalized spectral response of zeroth and first diffraction orders of NG, BFG, and LFG of period 560 nm, (**f**) self normalized spectral response of LFGs of various periods.

In order to explore the color selective mechanism, a normal grating with the same parameters as per the experiment is simulated using lumerical FDTD software (Ansys Optics 2022 R2.4, https://www.ansys.com/en-in/products/optics/fdtd). Figure 7a presents the simulated transmittance of a normal grating for various polarizations. For Transverse Electric (TE) polarization, a pronounced peak at 561 nm can be observed. In Transverse Magnetic (TM) polarization, the peak intensity slightly decreases, with additional resonant conditions at 639 nm, 734 nm, and 870 nm appearing. The black dotted circle shows the three dips and two peaks. The central dip at wavelength 639 nm corresponds to the plasmonic resonance excited between the air-Au interface. However, the other dips and peaks appear because the dip at 639 nm is in the middle of its transition to a peak. This transition depends on the metal thickness; it would become a peak for thicker metallic sheets^45^. In addition, the peak at wavelength 734 nm and dip at wavelength 870 nm correspond to the plasmonic resonance excited between the PMMA-Au and glass-Au interface, respectively. The electric and magnetic field profiles for TE and TM at the resonant peak of 561 nm are shown in Fig. 7b. Electric and magnetic fields are normalized separately. Inset images display the Poynting vector diagram in the yz-plane.

In the case of TE polarization, due to boundary conditions between PMMA and air, two opposite circulations of transverse Poynting vectors inside the PMMA groove are observed, where electric and magnetic field magnitudes are lower, as indicated by black arrows. This leads to saddle type singularities in the transverse Poynting vector pattern, as shown by the white dotted circles. Such singularities occur where either the electric or magnetic field vanishes^46^ (the magnetic field in the current scenario), giving rise to complex energy flow patterns within the structure. These singularities also lead to forward propagation of the transverse Poynting vector with high magnitude, where the electric field magnitude is high in the air region between two consecutive grooves, as shown by white arrows. This indicates energy transmission through the air gaps, contributing to the overall transmittance observed at the resonant wavelength, suggesting Mie type resonance through air gaps^46,47^. For TM polarization, no circulation of the transverse Poynting vector is observed; instead, transverse Poynting vectors are observed to diverge from air gaps where electric or magnetic fields are minimal (even zero), leading to focal type singularities. These singularities also lead to forward propagation of the transverse Poynting vectors in the PMMA grooves, as shown by white arrows, suggesting Mie resonance^46,48^ but through the grooves. Figure 7c shows the electric field, magnetic field, and transverse Poynting vector diagrams for LFG with charge 2 and 7 at the center of grating, at wavelength 561 nm (on resonance) and 500 nm (off-resonance). As LFG has varying duty cycles and curves within the structure, along the grating vector both the Mie resonances appear simultaneously at 561 nm wavelength in TE polarization, as shown in the first column, and only Mie resonance through the groove is observed in TM polarization, as shown in the second column. For a wavelength of 500 nm, the strength of the electric field, magnetic field, and transverse Poynting vector is reduced, as shown in the second last and last column of Fig. 7c for TE and TM polarization respectively, which indicates the absence of resonance.

Overall, the observed energy flow patterns and field distributions suggest the presence of Mie resonances that dominate over plasmonic resonances, and Mie resonance for TE polarization is higher than the Mie resonance for TM polarization. Other resonances, such as electric dipole resonance, magnetic dipole resonance, Fabry–Perot resonance^49,50^, guided mode resonance^51,52^, Bragg resonance^53^, Fano resonance^54,55^, Bloch mode resonance^56^, hyperbolic resonance^57^, magneto-optical resonance^58^, and bound state in continuum^59^ found in the literature, which could be possible in normal gratings. The electric and magnetic dipole resonances are not feasible in the proposed structure because, although the transverse Poynting vectors circulate (energy transferred to the mode), both the electric and magnetic fields are too weak to support the resonance^60^. The guided mode, Bloch mode, and Bragg resonances are also not feasible because no guided transverse Poynting vectors are along the grating vector directions^51–53,56,61^. There is no strong electric and magnetic field confinement forming node and antinode inside the groove in both TE and TM cases; hence, Fabry–Perot resonance is not feasible^49,50^. PMMA is not a hyperbolic or magnetic material in the visible region; hence, hyperbolic and magneto-optical resonance is not feasible^62^. Bound states in the continuum are also not feasible because no bound energy flow is observed^59^.

Figure 7d maps the wave vectors associated with the grating structure along with the color selectivity in various orders. The wave number of light ($k_{\text {0th}}$) under the resonance condition along the transmission zeroth diffraction order can be given by Eq. 7:

7 $$\begin{aligned} k_{\text {0th}} = \frac{2 \pi }{\lambda _{\text {0th}}}n_{\text {eff}}, \end{aligned}$$

Here, the resonant wavelength along the transmission zeroth diffraction order is ($\lambda _{\text {0th}}$) and the effective refractive index is ($n_{\text {eff}}$). The grating period $\Lambda$ is related to the reciprocal of the grating wave number $k_\text {g}$ by Eq. 8:

8 $$\begin{aligned} k_{\text {g}} = \frac{2 \pi }{\Lambda }. \end{aligned}$$

The wave number ($k_{\text {1st}}$) associated with wavelength ($\lambda _{\text {1st}}$) along the first diffraction order can be written using Pythagoras’ theorem, as shown in Eq. 9:

9 $$\begin{aligned} k_{\text {1st}} = \frac{2 \pi }{\lambda _{\text {1st}}}n_{\text {eff}} = \sqrt{k_{\text {g}}^2 + k_{\text {0th}}^2}. \end{aligned}$$

Hence, using Eqs. 7 to 9, $\lambda _{\text {1st}}$ can be expressed as Eq. 10:

10 $$\begin{aligned} \lambda _{\text {1st}} = \frac{1}{\sqrt{\left( \frac{1}{\lambda _{\text {0th}}}\right) ^2 + \left( \frac{1}{\Lambda n_{\text {eff}}}\right) ^2}}. \end{aligned}$$

Equation 10 suggests the blue shift in $\lambda _{\text {1st}}$ relative to the $\lambda _{\text {0th}}$ due to the interplay between the grating period and diffraction order. Similar spectral shift can also be observed experimentally as shown in Fig. 7e. The diffraction spectral responses shown in Fig. 7e were obtained for zeroth and first diffraction orders across normal, BFG, and LFG gratings. These spectral responses are captured considering (TE + TM)/2 polarization configuration under the crossed polarizer and analyzer setup to capture only resonance responses (crossed polarization microscopy technique^44^). These spectral responses are self normalized by dividing each data point by its maximum intensity values to compare only spectral shifts.

The zeroth order spectral response of the normal grating and BFG are nearly identical as shown in Fig. 7e. The first order spectral response of the normal grating, BFG, and LFG overlap, showing consistent diffraction characteristics. However, the first order spectral responses are blue shifted compared to the zeroth order spectral responses for each grating following the Eq.  10. In addition, the LFG exhibits a distinct zeroth order spectral response positioned between the responses of zeroth and the first orders of the normal or BFG grating. The spectral shift of the zeroth order of LFG can be attributed to the larger period of moiré pattern, which also follows the Eq. 10.

Figure 7f shows the self normalized experimentally obtained spectral response of LFGs with varying periods (520 nm, 560 nm, and 600 nm). Red shifts in the zeroth and first diffraction orders for increasing periods can be observed, which also follow Eq. 10. Overall, the on-axis structured beam is generated in the zeroth order using moiré pattern formed by overlapping two BFGs of different topological charges. The formed moiré pattern has a larger period, and by incorporating Mie resonance, the on-axis structured beam can be generated in visible region. All the 3D images are created using Blender 4.0, https://www.blender.org and microsoft powerpoint 2021, https://www.microsoft.com/en-in/microsoft-365/powerpoint.

## Conclusion

We have demonstrated a novel method for generating on-axis structured beams at the zeroth order of a diffraction grating using moiré-patterned binary gratings. By logically overlapping two binary forked gratings, we achieved the formation of a moiré pattern, which can be used as an alternative to spatial phase manipulation for on-axis structured beam generation. Simulations and experiments confirm the generation of an on-axis structured beam, whose shape is governed by the topological charges of the overlapped gratings. We have demonstrated the on-axis structured beam generation via a reconfigurable spatial light modulator as well as a standalone fabricated subwavelength grating to show its versatility. Furthermore, we introduced color-selective on-axis structured beam generation by incorporating Mie resonance in standalone metallo-dielectric moiré gratings, with experimental results showing that color selectivity depends on the grating period. This approach provides a scalable and reconfigurable pathway for structured beam generation, offering new possibilities for structured light applications.

## Methods

### Simulation method

The simulations are performed using Ansys Lumerical FDTD software (Ansys Optics 2022 R2.4, https://www.ansys.com/en-in/products/optics/fdtd). The refractive indices of Au and glass are taken from the software’s library as ‘Au-Johnson and Cristy’ and ‘${\text{SiO}}_{2}$-Palik’, respectively. The refractive indices of polymethyl methacrylate (PMMA) are taken from M. N. Polyanskiy et al.^63^. Periodic boundary conditions are applied in positive and negative X and Y directions, and perfectly matched layers are taken in positive and negative Z directions. The minimum mesh size is taken as 0.1 nm during the simulations.

### Fabrication method

A cleaned glass sample is first coated with 2 nm Cr and 15 nm Au using the electron beam evaporation method. Metal coated samples are spin coated using PMMA A4 at a 3000 rpm spin speed. Following that, PMMA coated samples are patterned using electron beam lithography. The acceleration voltage is maintained at 30 KV, and the aperture is kept at 10 $\upmu$m during patterning with the dose range of 250–600 $\upmu$C/cm^2^. Patterned samples are developed using the MIBK: IPA (1:3) solution.

## Data Availability

The data generated in this paper are available upon reasonable request from the corresponding author.
